# Effectiveness and Acceptability of Cognitive Behavioral Therapy and Family Therapy for Gaming Disorder: Protocol for a Nonrandomized Intervention Study of a Novel Psychological Treatment

**DOI:** 10.2196/56315

**Published:** 2024-08-16

**Authors:** Per Bore, Sara Nilsson, Mitchell Andersson, Kajsa Oehm, Joel Attvall, Anders Håkansson, Emma Claesdotter-Knutsson

**Affiliations:** 1 Section for Psychiatry, Department of Clinical Sciences Lund Faculty of Medicine Lund University Lund Sweden; 2 Clinical addiction research unit Malmö Addiction Center Region Skåne Malmö Sweden; 3 Child and Adolescent Psychiatry Regional Outpatient Care Region Skåne Lund Sweden

**Keywords:** gaming disorder, psychological treatment, CBT, cognitive behavioral therapy, family therapy, effectiveness, acceptability, gaming, addiction, mixed method design, video games, leisure activity, Sweden, young adult, teenager, internet gaming disorder

## Abstract

**Background:**

Gaming disorder (GD) is a new official diagnosis in the *International Classification of Diseases, 11th Revision*, and with its recognition, the need to offer treatment for the condition has become apparent. More knowledge is needed about the type of treatment needed for this group of patients.

**Objective:**

This study aims to evaluate the effectiveness and acceptability of a novel module-based psychological treatment for GD based on cognitive behavioral therapy and family therapy.

**Methods:**

This study is a nonrandomized intervention study, with a pretest, posttest, and 3-month follow-up design. It will assess changes in GD symptoms, psychological distress, and gaming time, alongside treatment satisfaction, working alliance, and a qualitative exploration of patients’ and relatives’ experiences of the treatment.

**Results:**

This study started in March 2022 and the recruitment is expected to close in August 2024.

**Conclusions:**

This study evaluates the effectiveness and acceptability of a psychological treatment for patients with problematic gaming behavior and GD. It is an effectiveness trial and will be conducted in routine care. This study will have high external validity and ensure that the results are relevant for a diverse clinical population with psychiatric comorbidity.

**Trial Registration:**

ClinicalTrials.gov NCT06018922; https://clinicaltrials.gov/study/NCT06018922

**International Registered Report Identifier (IRRID):**

DERR1-10.2196/56315

## Introduction

### Background

Playing video games is a common leisure activity, with 68% of those aged 13-16 years and 55% of those aged 17-18 years in Sweden reporting that they play regularly [1]. Among adults, 49% of men and 37% of women have played video games in the past 12 months, of whom 13% played daily [2]. While video gaming is a source of enjoyment for many, some individuals develop problems related to their gaming that negatively impact their well-being and everyday functioning. The research focusing on problematic gaming behavior, its impact on well-being and daily functioning, and potential treatments is still in its early stages of development.

In 2015, internet gaming disorder (IGD) was included in the *Diagnostic and Statistical Manual of Mental Disorders, Fifth Edition* (*DSM-V*) as a potential diagnosis in the section for disorders requiring further research [3]. In 2018, gaming disorder (GD) was included as an official diagnosis in the *International Classification of Diseases, 11th Revision* (*ICD-11*) [4]. GD is defined by a lack of control over one’s gaming behavior, prioritizing it over other activities, and continuing to play despite negative consequences, which results in increased psychological distress or problems with daily functioning. GD as a psychiatric disorder has been criticized by researchers who are concerned about the risk of medicalizing normal behavior, stigmatizing gaming, and pathologizing children’s everyday activities. They argue that sufficient evidence does not yet exist and that the criteria developed are overly based on other addiction disorders [5].

The global prevalence of GD is estimated to be 0.5% to 15% [6] and varies across studies and regions, with higher reported prevalence in Asia [7]. In a Swedish survey study, the prevalence rate was estimated to be 1.2% [8]. A meta-analysis of prevalence studies with a total of 226,247 individuals showed a global prevalence of 1.96% in all age groups; however, most of the included studies focused on adolescents and young adults [9]. There is high comorbidity with other psychiatric diagnoses, such as depression, anxiety, and obsessive-compulsive disorder [10-12], as well as neuropsychiatric conditions such as attention-deficit/hyperactivity disorder (ADHD) and autism spectrum disorder (ASD) [13,14]. Individuals with GD also demonstrate a heightened risk for suicidal ideation, sleep disturbances, poor academic performance, weaker emotional regulation, and poorer executive functioning [15-19]. Similar patterns have been observed in a Swedish context, where individuals who engaged heavily in gaming, both highly engaged gamers and gamers with problematic gaming behavior, experience loneliness and psychological distress [8,20]. In a 4-year longitudinal study, Hygen et al [21] observed no direct correlation or causation between IGD and mental health problems in children. They suggest that the concurrent emergence of these problems can be attributed to shared underlying factors.

GD is most common among male adolescents and young adults [9]. Time spent gaming is a weak predictor of symptoms of GD [22]. Research instead highlights gaming motives as crucial predictors of GD. Gaming used as an escape or coping mechanism is notably linked to severe problematic gaming behavior [23]. Several social factors are associated with GD in children and adolescents. Studies have shown that poorer quality relationships in the family are associated with increased severity of problem gaming [24]. A meta-analysis by Coşa et al [25] that investigated the parental factors for IGD showed that the lack of parental involvement and supervision, as well as family conflict and inadequate social support, are correlated with IGD. Paradoxically, some studies showed that overly strict restrictions can sometimes exacerbate the problem [25].

To date, the majority of studies have focused on various forms of cognitive behavioral therapy (CBT) as potential treatments for GD, and the results suggest that CBT is effective for GD [26-28]. Important studies on CBT for GD include CBT for internet addiction [29], CBT combined with family therapy (FT) [30], CBT combined with psychoeducation for parents [31], short-term treatment for internet and computer game addiction (STICA) [32], *Professioneller Umgang mit technischen Medien* (Professional Use of Technical Media; PROTECT+) [33], and relapse prevention [34]. Kim et al [28] conducted a network meta-analysis and found that despite the scarcity of intervention studies incorporating FT for GD, its combination with CBT may be a promising way to maximize treatment efficacy. Bonnaire et al [35] argue that it is important to involve parents in the treatment of GD because several studies link GD to the family environment and parent-child relationship. Multidimensional FT has been tested in a randomized controlled trial (RCT) for adolescents with IGD [36]. In comparison to FT treatment as usual, there were no significant differences between groups, although there were only 42 participants. Nonetheless, the whole group improved significantly from baseline to 12 months after treatment.

In conceptualizing our psychological treatment, we draw upon self-determination theory, which posits that 3 intrinsic needs—relatedness, competence, and autonomy—motivate behavior [37]. Research suggests that games proficiently satisfy these needs [38]. Given that individuals with GD often engage in gameplay to escape or cope, it may indicate a deficiency in need fulfillment within their real-world interactions, thereby further intensifying the allure of gaming [23,39]. We propose that these individuals have an imbalance within their motivational systems: an overactive threat system that deters engagement with the world outside gaming, coupled with a reward system tuned to gaming incentives. It is therefore important to strengthen individuals’ capabilities in meeting their needs in activities outside of gaming. Furthermore, considering the prevalence of gaming issues among adolescents, it is crucial to acknowledge the developmental and familial context in which problematic gaming behaviors manifest [24]. Individuals with GD often face difficulties in addressing their problematic behavior independently, which emphasizes the necessity of developing a systemic family-inclusive approach to treatment [35,40].

There is a call for more RCTs of psychological treatments for GD [26]. While efficacy trials offer high internal validity due to their controlled environments, they often deviate from clinical practice, which decreases the generalizability and applicability of the findings. Meanwhile, RCTs may not reflect real-world scenarios, as patients with comorbidities are often excluded and clinicians are directed to adhere strictly to a manual and limit parallel interventions [41]. Effectiveness trials, in contrast, evaluate treatments in standard clinical practices, embracing a broader patient demographic and intervention flexibility. This approach increases the relevance and generalizability of the findings. Meta-analyses suggest that, while efficacy trials often report larger treatment effects, the outcomes from effectiveness trials may be more representative of what clinicians can expect in their practice [42,43]. Our objective was to develop and evaluate a treatment within the local health care system to ensure its practical applicability in that setting, which was the reason we chose to carry out an effectiveness trial.

Considering the promise of combining CBT with FT to enhance the effects of psychological treatment, we aim to develop and evaluate the effectiveness of a novel module-based psychological treatment in a naturalistic setting that integrates interventions from CBT and FT for GD.

### Aims and Objectives

This 1-group, nonrandomized intervention study aims to evaluate the effectiveness and acceptability of a novel psychological treatment for GD offered to patients recruited at the outpatient clinic Gamingprojektet Maria Malmö in Sweden. The specific objectives of this study are, first, to evaluate the effectiveness of the psychological treatment in terms of changes in symptoms of GD, psychological distress, and time spent gaming. The second objective is to evaluate the acceptability of the treatment in terms of treatment satisfaction and working alliance. In addition to this objective, a qualitative study will be conducted focusing on exploring patients’ and relatives’ experience of the psychological treatment. The third objective is to explore how clinicians use and relate to a manual for psychological treatment of GD. It will be explored by evaluating which modules clinicians use in the treatment, how many sessions they spend on each module, and the total number of sessions required for each treatment.

## Methods

### Study Design and Setting

This study has a single group with a pretest, posttest, and 3-month follow-up design, as well as measures at each session. It is conducted in routine care and the participants are treatment-seeking patients. This study will be carried out at a single site, Gamingprojektet Maria Malmö, which is an outpatient clinic for problematic gaming behavior and GD in Skåne, Sweden. The participants will be offered a module-based psychotherapy that is a combination of interventions from CBT and FT. The treatment will be given by psychologists with a 5-year education in clinical psychology and psychotherapy, social workers with 1-year training in psychotherapeutic counseling, and psychotherapists with 2.5 years training in psychotherapeutic counseling. The treatment can be given as an individual treatment with the patient, a family treatment with only the parents, or a family treatment with both the parents and the patient. Most treatments will be conducted with 1 clinician per treatment, but exceptions can be made if there is a high level of conflict between the patient and the parents. Assessment points include baseline, which is the assessment period; the start of therapy (pretreatment period); the end of therapy (posttreatment period); and the follow-up after 3 months. In addition to these quantitative measures, we will also conduct qualitative interviews with patients and relatives about their views on the outcome, acceptability, and eventual negative experiences of the treatment, which will be presented in a separate paper.

**Figure 1:**
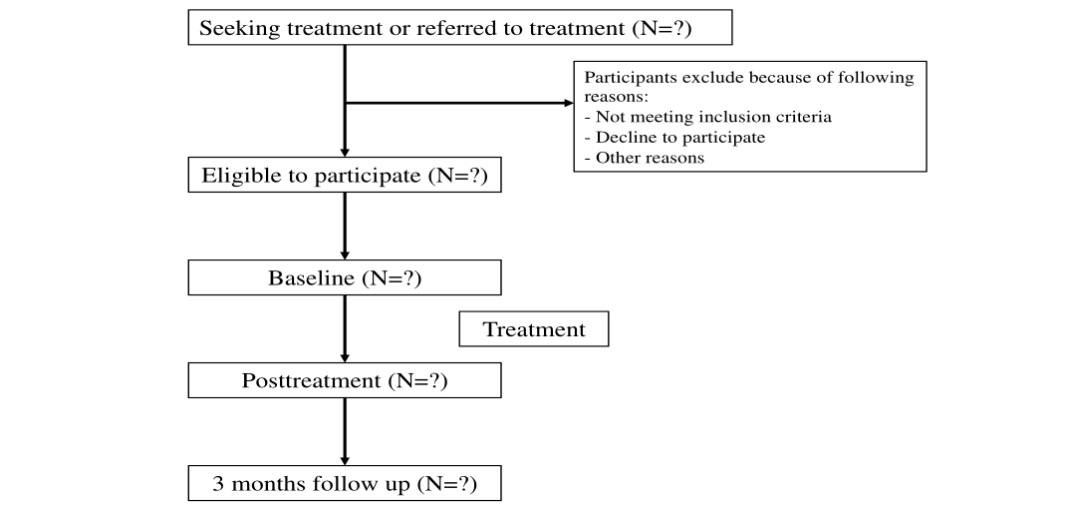
Recruitment and flow diagram (TRENDS). TRENDS: Transparent Reporting of Evaluations With Nonrandomized Designs.

A Transparent Reporting of Evaluations With Nonrandomized Designs 2004 flow diagram [44] of this study’s design is shown in Figure 1. This study is registered with ClinicalTrials.gov (NCT06018922). Recruitment started March 17, 2022, and will close on August 30, 2024.

### Recruitment

All patients who seek treatment or are referred to Gamingprojektet for GD treatment will be invited to participate in this study. The first patient was recruited in March 2022, and extensive work has been conducted to spread information about the clinic, both directly to the citizens by paid advertising on social media, public transport, billboards, and newspaper articles to reach inhabitants of Skåne county, and a campaign directed at professionals in the public health care system, in the school system, and social services. Patients come to the clinic either via self-referral or through referral from health care practitioners, schools, and social services. The health care system in Sweden is mostly government-funded, and all visits to this clinic are free of charge.

### Eligibility Criteria

This study aims to conduct a naturalistic effectiveness trial designed to include a representative group of patients; hence, inclusion and exclusion criteria are minimal. The inclusion criteria for participation include (1) being aged 13 years or older; (2) seeking treatment for problematic gaming behavior or GD as a patient or as a parent on behalf of their child; and (3) the patient’s gaming is affecting their well-being, daily functioning, or creating conflicts in the family.

The exclusion criteria for participation are (1) somatic or psychiatric disease that is contraindicating or severely complicates the implementation of the intervention (eg, ongoing psychotic, manic episode, or neuropsychiatric condition with severe disability) and in which case the patient is referred on from the clinic, and (2) the patient or parent (if the patient is aged younger than 15 years) is not able to read and communicate proficiently in Swedish.

### Treatment

The treatment that is studied is a novel psychological treatment developed by the researchers specifically for GD and is a combination of interventions from CBT and FT. The treatment is planned for approximately 15 sessions. However, it can be completed earlier if the patient chooses or extended beyond if the patient and clinician deem it necessary. The treatment is module-based with 10 individual modules and 7 family modules. The aim is to adapt each therapy and choose several modules, somewhere between 1 and 6 modules, depending on the patient’s age, family system, functioning, and case formulation. The modules have been developed by reviewing common interventions from CBT and FT, as well as other psychological treatments for GD. The modules are described in Textboxes 1 and 2.

**Individual modules.**

1. *Alternative activities to gaming (behavioral activation)*: helping the patient engage in other activities in addition to gaming, and problem-solving in session to increase the likelihood of engaging in other activities.
2. *Daily structure*: helping the patient create a clear and sustainable daily routine. For example, waking time, when to eat, when to do chores, and when to go to bed.
3. *Basic needs—nutrition, exercise, and sleep*: assisting the patient in developing better habits by providing psychoeducation about food, exercise, and sleep and planning together to change habits related to these basic needs.
4. *Impulse control*: identifying triggers for gaming, learning to delay acting on impulses, distracting oneself, and finding other behaviors that fulfill similar functions.
5. *Emotion regulation*: providing patients with psychoeducation about emotions, practicing recognizing their emotions, reflecting on how to manage strong emotions, and then practicing using these strategies at appropriate times.
6. *Working with thoughts*: working with the patient to relate more freely to their thoughts, not seeing them as truths and thereby reducing their reactions to them. This involves psychoeducation about thoughts, practicing how to approach them in therapy, and planning worry time.
7. *Social anxiety*: providing psychoeducation about common thoughts and mechanisms in social anxiety and exposure to situations where these thoughts and anxiety arise.
8. *Procrastination*: identifying procrastination behaviors and practicing strategies to manage these behaviors.
9. *Relationships and conflict management*: communication exercises and how the patient can handle conflicts with loved ones more helpfully.
10. *Problem-solving*: teaching the patient a step-by-step problem-solving strategy.

**Family modules.**

1. *Psychoeducation about gaming*: parents receive information about games, how they are played, and common motives for gaming. The purpose is to make parents more curious about gaming, focus more on the positive aspects of gaming, and understand how their children are gaming.
2. *Alternative activities to gaming*: helping parents engage their children in activities other than gaming, a form of behavioral activation. Specifically, starting with activities that children want to do and that can fulfill similar needs as gaming. Further, discussing the “activity plate model,” that is, what else they need in their lives besides gaming and how to incorporate it into their everyday life.
3. *Positive time together*: working to reduce conflicts in the family and creating more positive time together free from demands and conflict. This includes trying to avoid unnecessary conflicts, practicing positive communication, and acknowledging things that work.
4. *Demands and capabilities*: often, children have difficulties that parents are unaware of, or parents generally set higher demands on children than they can handle which increases the risk of the child turning to gaming to avoid the demands. We work to help the family to identify the right level of demands, and what support the patient needs to succeed in the tasks given to them.
5. *Making agreements in the family*: providing information and practicing together with the family on how to make agreements together and set boundaries. It is essential for children to feel listened to and participate in the discussion, even though parents may have the final say. This is performed by practicing concrete steps in making agreements together, first in the session together and then the family does it by themselves.
6. *Setting boundaries with emotional validation*: practicing how parents handle situations where they need to set boundaries for their children. The focus here is on becoming aware of their own and their children’s feelings and validating the children’s emotional experiences. The aim is to stay present with the children’s emotions without trying to problem-solve, eliminate the feelings, or escalate the conflict.
7. *Conflict management*: discussing how to handle situations where emotions become overwhelming in the family system. Often, it involves temporarily stepping back from the situation and revisiting the situation at a later point.

### Outcome Measures

This study consists of children, adolescents, and adults, as well as their parents. The patients will have different outcome measures depending on whether they are children (aged 13-15 years) or adults (aged 16 years and older). When the patient is a child, some outcome measures will also be collected from parents. An overview of outcome measures and assessment points is shown in Tables 1 and 2.

**Table 1:** Measures’ and measurements’ points for adults, aged 16+ years.

Measure	Outcome	Assessment	Treatment period			
		Baseline	Pretreatment	Weekly	Posttreatment	Month 3
CORE-OM 34^a^	Primary	✓	✓		✓	✓
GDT^b^	Primary	✓			✓	✓
IGDS9-SF^c^	Primary	✓			✓	✓
Time spent gaming	Primary	✓	✓	✓	✓	✓
How do you feel?	—^d^			✓		✓
Demographic	—	✓				
MINI^e^ interview	Secondary	✓			✓	
GAF^f^	Secondary	✓			✓	
NODS-PERC^g^	Secondary	✓			✓	
Social media disorder	Secondary	✓			✓	
Game motives	Secondary	✓			✓	
Emotion regulation	Secondary	✓			✓	
Alcohol use	Secondary	✓			✓	
Substance use	Secondary	✓			✓	
Harm severity of gaming	Secondary	✓			✓	
Working alliance	Acceptability					
Treatment satisfaction	Acceptability					

^a^CORE-OM 34: Clinical Outcomes in Routine Evaluation–Outcome Measure 34 questions.

^b^GDT: Gaming Disorder Test.

^c^IGDS9-SF: Internet Gaming Disorder Scale–Short-Form.

^d^Not applicable.

^e^MINI: Mini International Neuropsychiatric Interview.

^f^GAF: Global Assessment of Functioning.

^g^NODS-PERC: National Opinion Research Centre Diagnostic Screen for Gambling Disorders–Preoccupation, Escape, Risked Relationships, and Chasing.

**Table 2:** Measures’ and measurements’ points for children, aged 13-15 years.

Measure	Outcome	Assessment	Treatment period			
		Baseline	Pretreatment	Weekly	Posttreatment	Month 3
RCADS^a^-child	Primary	✓	✓		✓	✓
RCADS-parent	Primary	✓	✓		✓	✓
GDT^b^	Primary	✓			✓	✓
IGDS9-SF^c^	Primary	✓			✓	✓
GAIT^d^-parent	Primary	✓			✓	✓
Time spent gaming	Primary	✓	✓	✓	✓	✓
How do you feel?	—^e^			✓		
Demographic	—	✓				
MINI^f^-kid interview	Secondary	✓			✓	
CGAS^g^	Secondary	✓			✓	
NODS-PERC^h^	Secondary	✓			✓	
Social media disorder	Secondary	✓			✓	
Game motives	Secondary	✓			✓	
Emotion regulation	Secondary	✓			✓	
Alcohol use	Secondary	✓			✓	
Substance use	Secondary	✓			✓	
Harm severity of gaming	Secondary	✓			✓	
Working alliance	Acceptability				✓	
Treatment satisfaction	Acceptability				✓	

^a^RCADS: Revised Child Anxiety and Depression Scale.

^b^GDT: Gaming Disorder Test.

^c^IGDS9-SF: Internet Gaming Disorder Scale–Short-Form.

^d^GAIT: Gaming Addiction Identification Test.

^e^Not applicable.

^f^MINI: Mini International Neuropsychiatric Interview.

^g^CGAS: Children’s Global Assessment Scale.

^h^NODS-PERC: National Opinion Research Centre Diagnostic Screen for Gambling Disorders–Preoccupation, Escape, Risked Relationships, and Chasing.

### Primary Outcome Measures

#### Overview

The primary outcome measures are symptoms of GD, psychological distress, and time spent on gaming in the last week.

#### Adults

Symptoms of GD are measured with both the Gaming Disorder Test (GDT) [45] and the Internet Gaming Disorder Scale–Short-Form (IGDS9-SF) [46]. The GDT consists of 4 questions that cover the criteria for GD in *ICD-11* and is answered on a 5-point Likert scale, from 1 to 5. The total score is 20 and the suggested cutoff is that all questions are answered with 4 or 5. The IGDS9-SF consists of 9 questions that cover the criteria for IGD in the *DSM-V*, with an additional question added about craving, and is answered on a 5-point Likert scale, from 1 to 5. The total score for the original IGDS9-SF questions is 45. The suggested cutoff point based on an Italian clinical sample is 21 [47]. Psychological distress is measured by the Clinical Outcomes in Routine Evaluation–Outcome Measure 34 questions (CORE-OM 34). The CORE-OM 34 contains 34 questions about subjective well-being, symptoms (anxiety, depression, and physical problems), life functioning, and risk of suicide [48]. It is answered on a 5-point Likert scale, from 1 to 5. The last primary outcome measure is how much time the patient has spent on gaming in the last week, where the patient themselves approximates how much time they have spent gaming.

#### Children

Symptoms of GD are measured with the GDT, IGDS9-SF, and Gaming Addiction Identification Test [49]. The Gaming Addiction Identification Test is a parent assessment with 17 questions that is answered on a 5-point Likert scale, from 1 to 5. It also contains a question about the estimated time spent gaming per day. Psychological distress is measured by the Revised Child Anxiety and Depression Scale (RCADS) [50] child and parent self-report. The RCADS contains 47 items assessed on a 5-point Likert scale, from 1-5, for children and parents, covering subscales for social phobia, panic disorder, general anxiety disorder, obsessive-compulsive disorder, separation anxiety, and depression. Scores are compiled into 2 totals: one for combined anxiety and depression, and another for exclusively anxiety-related symptoms. The RCADS is globally recognized in research and clinical settings for its strong psychometric reliability [50]. Lastly, the children estimate how much time they have spent gaming during the last week.

### Secondary Outcome Measures

#### Overview

The secondary outcome measures are psychiatric comorbidity, everyday functioning, emotional deregulation, problematic social media behavior, gaming motives, symptoms of problem gambling, alcohol use, substance use, harm severity of gaming, therapeutic alliance, and treatment satisfaction.

#### Adults

Psychiatric comorbidity is measured by semistructured interviews by a clinician with the Mini International Neuropsychiatric Interview which assesses common psychiatric diagnoses from the *DSM-V* and *International Classification of Diseases, 10th Revision* (*ICD-10*) [51]. Everyday functioning is measured by clinicians using the Global Assessment of Functioning [52], where the clinician assesses everyday function on a 1-100 scale. Emotional dysregulation is measured using the Difficulties in Emotion Regulation Scale-16 [53], which consists of 16 items. Problematic social media use is measured with the Bergen Social Media Addiction Scale, which contains 6 questions answered on a 5-point Likert scale, from 1 to 5, about salience, mood modification, tolerance, withdrawal, conflict, and relapse [54]. Gaming motives are assessed using the Motives for Online Gaming Questionnaire, which contains 27 questions answered on a 5-point Likert scale, from 1 to 5 [55]. The questionnaire assesses 7 motivational factors: escape, coping, competition, social, skill development, fantasy, and recreation. Problem gambling is measured with the National Opinion Research Centre Diagnostic Screen for Gambling Disorders–Preoccupation, Escape, Risked Relationships, and Chasing, consisting of 4 questions about gambling problems throughout one’s lifetime, focusing on preoccupation, chasing losses, escape, and the social consequences of gambling, answered on a binary scale. If the participant endorses any item, it indicates problem gambling [56]. To assess the level of alcohol consumption, we use the Alcohol Use Disorders Identification Test-Consumption scale, 3 questions, which focus on the frequency and quantity of alcohol consumption [57]. To measure substance use behavior, a drug identification list consisting of 10 common substances is given to the patients from which they indicate whether they have ever used said substance, and if so, how often. Gaming-related harm severity was assessed by questions about how the patient’s gaming impairs their ability to function in school or work, everyday situations at home, social activities, free time, and family life. The scale ranges from 0 (indicating no harm or minimal impact) to 10 (signifying severe harm) on how gaming impairs their function in the 5 parts of their everyday life. The therapeutic alliance is measured by the Working Alliance Inventory Short, which contains 12 questions about the alliance in the therapeutic relationship [58]. Further, 9 additional questions were developed by the research team and included regarding patient treatment satisfaction.

#### Children

In total, 2 of the instruments are different between the children and adults. Psychiatric comorbidity is measured by using the Mini International Neuropsychiatric Interview for Children and Adolescents [59]. Everyday functioning is measured by the clinician using the Children’s Global Assessment Scale [60], where clinicians assess everyday function on a scale from 1 to 100.

### Additional Measures

Data concerning demographics, what games they are playing, everyday gaming behavior, social life, family life, psychical health, sleep and eating habits, as well as symptoms of ASD and ADHD, are gathered at baseline to provide a comprehensive picture of the treatment-seeking sample. For the adults, ASD symptoms will be measured with Ritvo Autism and Asperger Diagnostic Scale 14 [61]. Symptoms of ADHD will be measured by the Adult ADHD Self-Report Scale part A, which contains 6 items, and it has been found that these questions are the most predictive of ADHD [62]. For children, patient-reported symptoms of ASD and ADHD are measured with Ritvo Autism and Asperger Diagnostic Scale 14 and Adult ADHD Self-Report Scale, respectively. Likewise, parent-reported child ASD and ADHD symptoms are measured using the Autism Quotient [63] and Swanson, Nolan, and Pelham [64] scales, respectively. After each treatment session, the patients will answer 4 questions: rating their psychological well-being on a 1-10 scale, reporting how much time they have spent gaming the last week, rating how much their psychological distress depends on their gaming on a scale of 1-10, and the reporting of how helpful they found this treatment session on a 1-10 scale. After treatment, the patients will be asked whether they have received any parallel interventions during the treatment and whether they will seek further help for their problems. Before treatment begins, the patients’ current medications will be documented. During the posttreatment assessment, any changes to their medications during the treatment will be recorded. These changes will be reported at the group level in the final paper.

### Sample Size

This study is performed in routine care and a naturalistic setting, which means that how many participants can be recruited is dependent on how many patients seek treatment at the said outpatient clinic. We aim to conduct semistructured interviews with at least 20 participants, both patients and parents, or until data saturation is reached.

### Statistical Analysis and Power Analysis

We will present descriptive statistics (eg, proportions, means, SD, and medians) and bivariate correlation measures for variables collected at baseline. To assess the overall effect of the treatment phase (baseline, postintervention, and follow-up) on primary (eg, GD symptom burden, psychological distress, and time spent gaming) and secondary continuous outcome measures, we will conduct repeated measures mixed-effect ANOVA with time as a fixed factor and patient as a random factor; post hoc pairwise comparisons with Bonferroni corrections will be conducted to determine differences in outcome variables across treatment phases. This approach is essentially equivalent to repeated measures ANOVA but will enable us to control individual variability and perform analyses including all cases provided each patient has 1 valid pre- or postoutcome value. In addition, we will conduct logistic mixed-effect model analyses to assess the effect of time on binary outcome measures (ie, symptom improvement).

All tests will be 2-sided with the α level set at .05. Based on previous CBT-based therapy studies for GD that indicate medium-to-large effect sizes from pre- to posttest for symptom burden, time playing, and mental health improvement [32], we estimate that a minimum of 28 participants would be required to achieve 80% power to detect such differences across all outcome measures (Cohen *f*=0.25). To accommodate a potential attrition rate of 30%, we will enroll a minimum of 37 participants for quantitative analyses. Follow-up univariate logistic regressions will be conducted to explore which baseline characteristics are predictive of participant symptom improvement, defined by subcutoff scores for the GDT, IGDS9-SF, and CORE-OM 34 follow-up. Planned analyses will be conducted apropos of missing data patterns and modified accordingly when needed. The results will be reported as means and odds ratios, respectively, with 95% CIs.

### Qualitative Component

To bolster our quantitative findings and refine our treatment model, our project will integrate a qualitative component. This phase will consist of semistructured interviews with patients and their relatives, exploring their thoughts and experiences of GD and the psychological treatment they underwent. The interviews will center on gaining insights into their comprehension of GD, their perceptions of the treatment’s effectiveness, its acceptability, and whether it appropriately addressed their needs. Furthermore, the interviews will probe into the mechanisms of change, exploring participants’ experiences with individual or family-focused aspects of the treatment.

Interviews will be conducted by staff with knowledge of qualitative interview methodology and will be recorded and transcribed verbatim. Initially, we will remove person identifiers and pseudonymize the data. Further, 2 researchers will independently acquaint themselves with the data and identify potential themes. These will then be jointly reviewed and defined, culminating in their formal naming and clarification. Quotes will be translated to English via a back-translation procedure in collaboration with an independent bilingual party to ensure accuracy.

### Ethical Considerations

This study is approved by the Swedish Ethical Review Authority (reference: 2021-06666-01, 2022-03-17). Subsequent amendments have been approved (reference: 2023-03112-02; 2023-03112-02, reference: 2023-03112-02; 2023-06-05, and reference: 2023-06393-02; 2023-11-08). We estimate that this study poses little or no risk for the participants, especially since this study is carried out in routine care. The patient’s choice to participate in the research will not impact the treatment they receive and they will go through the same assessment before and after treatment regardless of whether they take part in the research or not. All participants will give written consent to participate in this study and may withdraw at any point. For patients aged 13 and 14 years, the children will give assent and parents will give written consent.

### Participants’ Safety

This study is integrated into routine care, ensuring that standard clinical safety procedures are adhered to. There are weekly meetings where the clinicians discuss treatment progress and potential adverse events. The patients answer a questionnaire where they rate their psychological well-being at every treatment session, and the clinicians monitor these forms, which enables them to detect a change in the patients’ well-being. If a patient starts deteriorating in psychological distress, develops a new psychiatric disorder, or has emerging suicidal ideation, it will be addressed in the ongoing psychological treatment. If necessary, an experienced psychiatrist is available to adjust medication or assess the need for inpatient care.

### Data Management

Personal and identifiable data will be collected from patients. All collected data will be stored securely, with the physical documents being stored in locked cabinets and digital data behind firewalls and only be accessible by the clinicians and researchers. The data from all patients will be compiled in a database with study ID. This study ID will be recorded in a document, which will be stored separately from the physical and digital data and can only be accessed by 1 member of the research team.

### Dissemination

Findings will be published in peer-reviewed journals, at research conferences, and disseminated to the general public.

This study will be reported per the Transparent Reporting of Evaluations With Nonrandomized Designs statement. The primary objective is to compose a key scientific paper concentrating on the efficacy and acceptability of the treatment. Subsequent studies within the research project will include qualitative interviews and analysis to explore the experiences of both patients and their relatives with the treatment. Additional papers may also emerge, delving into predictors and moderators influencing treatment outcomes.

## Results

This study started on March 17, 2022, and recruitment is planned to end on August 30, 2024. The first results are expected in 2025.

## Discussion

### Principal Findings

This study will evaluate the effectiveness and acceptability of psychological treatment for patients with problematic gaming behavior and GD about symptoms of GD and psychological distress and time spent gaming. This study will be a 1-group, pretest-posttest with a follow-up design including at least 37 patients undergoing psychological treatment at an outpatient clinic specialized in GD. The treatment is module-based and is based on CBT and FT. The treatment will be given as individual sessions with the patient sessions, family sessions, sessions with parents, or a combination of the three. The duration of the therapy provided to each patient can vary, ranging from 5 to 20+ sessions based on the case formulation of the patient and the progression of the therapy.

This study is important in several aspects: this study has a high external validity since it is conducted in routine care. Due to that, this study ensures that the results are relevant for a diverse clinical population with patients with high comorbidity with other psychiatric conditions. The treatment consisting of standard CBT and FT interventions not only roots this study in well-established therapeutic techniques but also indicates that, should the results be positive, the treatment protocol can easily be adopted by other clinics. Moreover, this study’s origins in actual clinical practice underscore its commitment to real-world effectiveness, emphasizing the value of learning directly from clinical experiences and spreading that knowledge.

There are feasibility and exploratory elements to this study. The patients answer questions about satisfaction and working alliance with the therapist at the posttreatment assessment. There are also planned qualitative interviews with the patients and relatives of the patients regarding their view of the treatment, important components of the treatment, if anything should change, and the patient-clinician relationship.

### Limitations and Strengths

The primary limitation of this study is its single-arm design, with 1 intervention group and no control group. This design makes it hard to draw any definitive conclusions about the effectiveness of the treatment, as opposed to potential influences from the patient–health care professional interactions or the natural course of the disorder’s reduction over time. However, we can compare our results with outcomes from other studies on treatment for GD. Several factors influenced our decision to pursue a single-group trial design. First, we anticipated recruitment challenges given that this study would be conducted at a newly established clinic. Ethical considerations also played a part; we felt it imperative to offer the best possible treatment to those seeking assistance, rather than withholding potential benefits for the sake of a control group. There is no established treatment for GD, and therefore, we could not have another treatment arm in this study. Due to recruitment challenges, in terms of few patients seeking treatment for GD and a lack of awareness about the newly opened clinic, we decided not to use a waiting list control group. Lastly, given this study’s exploratory nature, our objective was to evaluate the treatment in a real-world setting, examine how many sessions the clinicians often offer, and which of the modules are most often used.

### Conclusion

In summary, this study aims to evaluate a novel psychological treatment for GD in a naturalistic setting, using a 1-group pretest and posttest design.

## Abbreviations

ADHD: attention-deficit/hyperactivity disorder
ASD: autism spectrum disorder
CBT: cognitive behavioral therapy
CORE-OM 34: Clinical Outcomes in Routine Evaluation–Outcome Measure 34 questions
DSM-V: Diagnostic and Statistical Manual of Mental Disorders, Fifth Edition
FT: family therapy
GD: gaming disorder
GDT: Gaming Disorder Test
ICD-10: International Classification of Diseases, 10th Revision
ICD-11: International Classification of Diseases, 11th Revision
IGD: internet gaming disorder
IGDS9-SF: Internet Gaming Disorder Scale–Short-Form
PROTECT+: Professioneller Umgang mit technischen Medien (Professional Use of Technical Media)
RCADS: Revised Child Anxiety and Depression Scale
RCT: randomized controlled trial
STICA: short-term treatment for internet and computer game addiction

## Data Availability

The data sets generated or analyzed during this study are not publicly available because this study’s ethical review does not allow for study data to be in a public repository. Requests to access the data sets should be addressed to PB.
